# Aggressive and self-harming behaviour among psychiatric inpatients in a specialised prison hospital: a retrospective cohort study

**DOI:** 10.3389/fpsyt.2026.1902603

**Published:** 2026-08-12

**Authors:** Alexander Blees, Angela Luzia Herscheid, Sharon Jakobowitz, Peter Seidel, Roman Köster, Annette Opitz-Welke

**Affiliations:** 1 Institute of Forensic Psychiatry, Charité-Universitätsmedizin Berlin, Berlin, Germany; 2 Department of Psychiatry and Psychotherapy, Prison Hospital Berlin, Berlin, Germany; 3 Criminological Service for the Berlin Prison System and Social Services of the Judiciary, Berlin, Germany; 4 Department of Internal Medicine, Prison Hospital Berlin, Berlin, Germany

**Keywords:** aggressive behaviour, correctional psychiatry, prison psychiatry, schizophrenia, self-harm

## Abstract

**Background:**

Aggressive behaviour and self-harming behaviour are common psychiatric emergencies in correctional settings, yet little is known about the factors distinguishing these behavioural presentations in psychiatric inpatients. This study compared the sociodemographic and clinical characteristics of psychiatric inpatients presenting with aggressive or self-harming behaviour in a specialised prison hospital.

**Methods:**

In this retrospective cohort study, routinely collected data from 643 psychiatric inpatients admitted to the Berlin Prison Hospital (2010–2016) were analysed. Patients presenting with aggressive behaviour (n = 73) or self-harming behaviour (n = 60) were each compared with the remaining psychiatric inpatients using multivariable logistic regression.

**Results:**

Schizophrenia spectrum disorders were independently associated with lower odds of self-harming behaviour after multivariable adjustment. Parenthood was independently associated with lower odds of aggressive behaviour and higher odds of self-harming behaviour. In contrast, stress-related disorders and personality disorders were not independently associated with either behavioural presentation after adjustment.

**Conclusions:**

Aggressive and self-harming behavioural emergencies were associated with different clinical and sociodemographic characteristics among psychiatric inpatients in a specialised prison hospital. Parenthood emerged as the most consistent independent correlate of aggressive and self-harming behaviour presentation, whereas schizophrenia spectrum disorders were independently associated with lower odds of self-harming behaviour. These findings may contribute to behaviour-specific risk assessment and prevention strategies in correctional psychiatry.

## Background

Aggressive behaviour and self-harm are common behavioural emergencies in correctional settings and represent major challenges for both patient care and institutional safety. Both behavioural presentations are associated with considerable morbidity and frequently require coercive interventions, prolonged psychiatric treatment, and substantial healthcare resources. Whereas aggressive behaviour primarily threatens fellow prisoners, correctional officers, and healthcare professionals, self-harming behaviour is closely linked to suicide prevention and constitutes one of the strongest predictors of subsequent suicide. Suicide remains one of the leading causes of death during imprisonment worldwide, with incidence rates consistently exceeding those in age-standardised community populations, highlighting the importance of early identification and management of behavioural crises in correctional psychiatry (1–3).

People deprived of liberty experience substantially higher rates of mental disorders than the general population. Meta-analyses have consistently reported markedly increased prevalence rates of schizophrenia spectrum disorders, major depressive disorder, personality disorders, substance use disorders, and trauma-related disorders among incarcerated individuals (1, 4, 5). Consequently, specialised prison psychiatric services care for a highly vulnerable population in whom severe mental illness frequently coincides with acute behavioural crises. These crises rarely arise from psychiatric disorders alone. Rather, they reflect the interaction between individual vulnerability and prison-specific stressors such as deprivation of liberty, uncertainty regarding legal proceedings, social isolation, victimisation, and restricted autonomy. Recent evidence emphasises that self-harming behaviour in prison should be understood within its environmental and interpersonal context rather than as an isolated manifestation of psychiatric illness. Trauma exposure, neurodevelopmental disorders, acquired brain injury, institutional deprivation, and prison culture may all contribute to behavioural crises. These factors may also influence both clinical presentation and staff responses (2, 6, 7).

Beyond psychiatric disorders and prison-specific stressors, social connectedness has increasingly been recognised as an important determinant of mental health during imprisonment. Recent systematic reviews have shown that reduced social support, limited family contact, and loneliness are associated with poorer mental health among prisoners, and that family relationships may represent an important source of emotional support during incarceration (8, 9). In addition, reduced social support has been identified as a modifiable risk factor for self-harming behaviour in prison populations (2). However, it remains unclear whether routinely documented indicators of social connectedness, such as relationship status or parenthood, are associated with different behavioural emergencies among psychiatric inpatients in prison settings.

Traditionally, aggression and self-harming behaviour have been investigated as separate clinical phenomena. Aggression has primarily been studied from the perspective of institutional violence and forensic risk assessment, whereas research on self-harming behaviour has focused on suicide prevention and emotional dysregulation. However, growing evidence indicates that both behaviours share several vulnerability factors, including severe mental illness, impulsivity, substance use disorders, childhood adversity, traumatic experiences, and impaired emotion regulation (10). Recent research suggests that aggression and self-harming behaviour are interconnected through shared psychological and behavioural mechanisms rather than representing entirely independent phenomena. A network analysis demonstrated close associations between both behaviours via common pathways such as emotional dysregulation and psychiatric symptoms (11). Consistent with these findings, the concept of dual harm proposes that aggression towards other people and self-harming behaviour frequently co-occur and represent overlapping behavioural manifestations rather than entirely distinct clinical entities (12, 13).

Schizophrenia spectrum disorders are of particular relevance to the present study because they have been associated with both aggressive and self-harming behaviour. Although psychotic disorders have traditionally been linked to an increased risk of violence, recent evidence indicates that this association is substantially influenced by comorbid substance use disorders, previous violence, and adverse social circumstances rather than psychosis itself (14, 15). At the same time, self-harming behaviour is increasingly recognised as a common feature of schizophrenia spectrum disorders, with a recent meta-analysis reporting a lifetime prevalence of self-harming behaviour of approximately one-third among individuals with schizophrenia spectrum disorders (16).

Within correctional settings, behavioural crises are further shaped by environmental factors. Systematic reviews have identified previous self-harm, psychiatric disorders, suicidal ideation, solitary confinement, and reduced social support as major determinants of self-harming behaviour during imprisonment (2). Observational studies have likewise identified previous psychiatric treatment, remand detention, and a history of self-harm as important risk factors for suicide in prison populations (6, 17, 18). Collectively, these findings support a biopsychosocial model in which behavioural emergencies arise from the interaction between psychiatric illness and the correctional environment rather than diagnostic categories alone.

Despite considerable progress, important knowledge gaps remain. Most previous studies have investigated aggressive and self-harming behaviour separately or within heterogeneous prison populations. Comparatively little is known about psychiatric inpatients requiring acute treatment in specialised prison hospitals, although this population represents the most severely ill subgroup within correctional psychiatry. Evidence from specialised prison psychiatric services remains limited, especially in Germany, where the organisation of correctional healthcare differs from that of many other countries. Furthermore, little is known about how patients presenting with aggressive or self-harming behavioural emergencies differ from the broader population of psychiatric inpatients treated in specialised prison hospitals. Previous work from the Berlin prison system highlighted the substantial burden of self-harming behaviour and the need for improved identification of prisoners at increased risk of behavioural crises (19). However, it remains unclear whether these behavioural emergencies represent distinct clinical profiles requiring different prevention and treatment strategies.

The present study aimed to compare the sociodemographic and clinical characteristics of psychiatric inpatients presenting with aggressive behaviour or self-harming behaviour with those of the remaining psychiatric inpatient population. Furthermore, we examined whether the investigated sociodemographic and clinical characteristics, including primary psychiatric diagnoses, remained independently associated with each behavioural presentation after multivariable adjustment.

## Materials and methods

### Study design and setting

This retrospective cohort study analysed routinely collected data from the electronic medical records of psychiatric inpatients treated at the Department of Psychiatry and Psychotherapy of the Berlin Prison Hospital between January 2010 and December 2016.

The Berlin Prison Hospital is the central inpatient healthcare facility for all correctional institutions in the federal state of Berlin and provides specialised psychiatric care for incarcerated individuals requiring acute inpatient treatment. During the study period, only male prisoners were admitted to the psychiatric ward, as female prisoners requiring inpatient psychiatric treatment were managed in separate correctional facilities. Referrals originated from prison physicians and the hospital’s psychiatric consultation service. Psychiatric diagnoses were established by experienced prison psychiatrists according to the International Statistical Classification of Diseases and Related Health Problems, Tenth Revision (ICD-10).

### Participants

All psychiatric inpatient admissions during the study period were screened for eligibility using a routinely maintained electronic clinical database. Analyses were subsequently performed at the patient level to ensure statistical independence. For patients with repeated admissions, only one admission was selected. Among patients presenting with aggressive or self-harming behaviour, the admission corresponding to the first documented behavioural event was selected. For patients without documented behavioural emergencies, the first psychiatric admission during the study period was selected.

Patients were classified into four mutually exclusive groups:

- aggressive behaviour.
- self-harming behaviour.
- dual harm (co-occurring aggressive and self-harming behaviour).
- patients without documented behavioural emergencies.

Because only seven patients presented with dual harm, this group was included in the descriptive analyses but excluded from comparative analyses owing to insufficient statistical power for meaningful statistical inference.

### Variables

Clinical, sociodemographic, and detention-related information was extracted retrospectively from the electronic medical records of the index admission.

Sociodemographic variables included age, nationality, interpreter requirement, relationship status, and parenthood. Detention-related variables included detention status and previous imprisonment. Clinical variables included primary psychiatric diagnosis, psychiatric comorbidity, and behavioural presentation.

Primary psychiatric diagnoses recorded in the electronic medical records were established by experienced prison psychiatrists according to ICD-10. Psychiatric comorbidities were documented separately.

### Outcome definitions

Behavioural presentations were identified from routine clinical documentation completed by psychiatrists and multidisciplinary ward staff.

Self-harming behaviour comprised intentional self-directed injury or suicidal behaviour immediately preceding or occurring during psychiatric admission. This category included non-suicidal self-injury, suicidal ideation requiring psychiatric inpatient treatment, suicide attempts, and completed suicide.

Aggressive behaviour comprised verbal or physical aggression directed towards other persons or the institutional environment immediately preceding or occurring during psychiatric admission. Behavioural events included verbal threats, physical assaults, weapon-related incidents, and arson.

### Statistical analysis

Continuous variables are presented as means ± standard deviations (SD), whereas categorical variables are reported as frequencies and percentages. Continuous variables were compared using independent-samples t-tests. Categorical variables were compared using Fisher’s exact test because several contingency tables contained small expected cell counts.

Independent variables were selected *a priori* based on clinical relevance, theoretical plausibility, and evidence from previous literature, while also considering the observed group differences in the univariable analyses. To minimise the risk of model overfitting, only clinically meaningful and sufficiently frequent variables were entered into the regression models. Rare diagnostic categories and variables that lacked a consistent clinical or statistical rationale for inclusion were not considered.

Two multivariable binary logistic regression models were fitted to examine factors independently associated with aggressive behaviour and self-harming behaviour. The first model compared patients presenting with aggressive behaviour with all remaining psychiatric inpatients. The second model compared patients presenting with self-harming behaviour with all remaining psychiatric inpatients. The same independent variables (age, detention status, parenthood, schizophrenia spectrum disorders [ICD-10 F2], stress-related disorders [ICD-10 F4], and personality disorders [ICD-10 F6]) were entered simultaneously into both models using the enter method.

Cases with missing values for variables included in the respective regression model were excluded using complete-case analysis. Missing data were limited to parenthood (n = 14) and detention status (n = 2), whereas all psychiatric diagnostic variables were complete. Given the small proportion of missing data, complete-case analysis was considered an appropriate approach that avoided the additional assumptions required for data imputation.

Model calibration was assessed using the Hosmer–Lemeshow goodness-of-fit test, and explanatory performance was evaluated using Nagelkerke’s R². Results are reported as odds ratios (ORs) with corresponding 95% confidence intervals (95% CIs). Statistical significance was defined as a two-sided *p* < 0.05.

All statistical analyses were performed using IBM SPSS Statistics for Mac, Version 31.0.0.0 (IBM Corp., Armonk, NY, USA).

### Ethics approval and consent to participate

The study was approved by the Ethics Committee of Charité – Universitätsmedizin Berlin (approval number EA2/238/24; approval date: 19 November 2024) and conducted in accordance with the principles of the Declaration of Helsinki.

Owing to the retrospective study design and the use of routinely collected clinical data, the requirement for informed consent was waived by the Ethics Committee. In accordance with Section 17 (1) of the Berlin Data Protection Act, the use of personal data for scientific research without individual consent is permissible when obtaining consent would require disproportionate effort and the public interest in conducting the research outweighs the individual’s interest in excluding the processing of their data. These conditions were reviewed and approved by the Ethics Committee as part of the approval process.

Because the study involved a vulnerable prison population, approval for the use of the data was also obtained from the Criminological Service of the Berlin Prison System. All data were analysed retrospectively in anonymised form.

## Results

### Study population

Between January 2010 and December 2016, 866 psychiatric inpatient admissions were recorded at the Department of Psychiatry and Psychotherapy of the Berlin Prison Hospital. After restricting the dataset to one admission per individual, the final study cohort comprised 643 unique patients.

**Figure 1:**
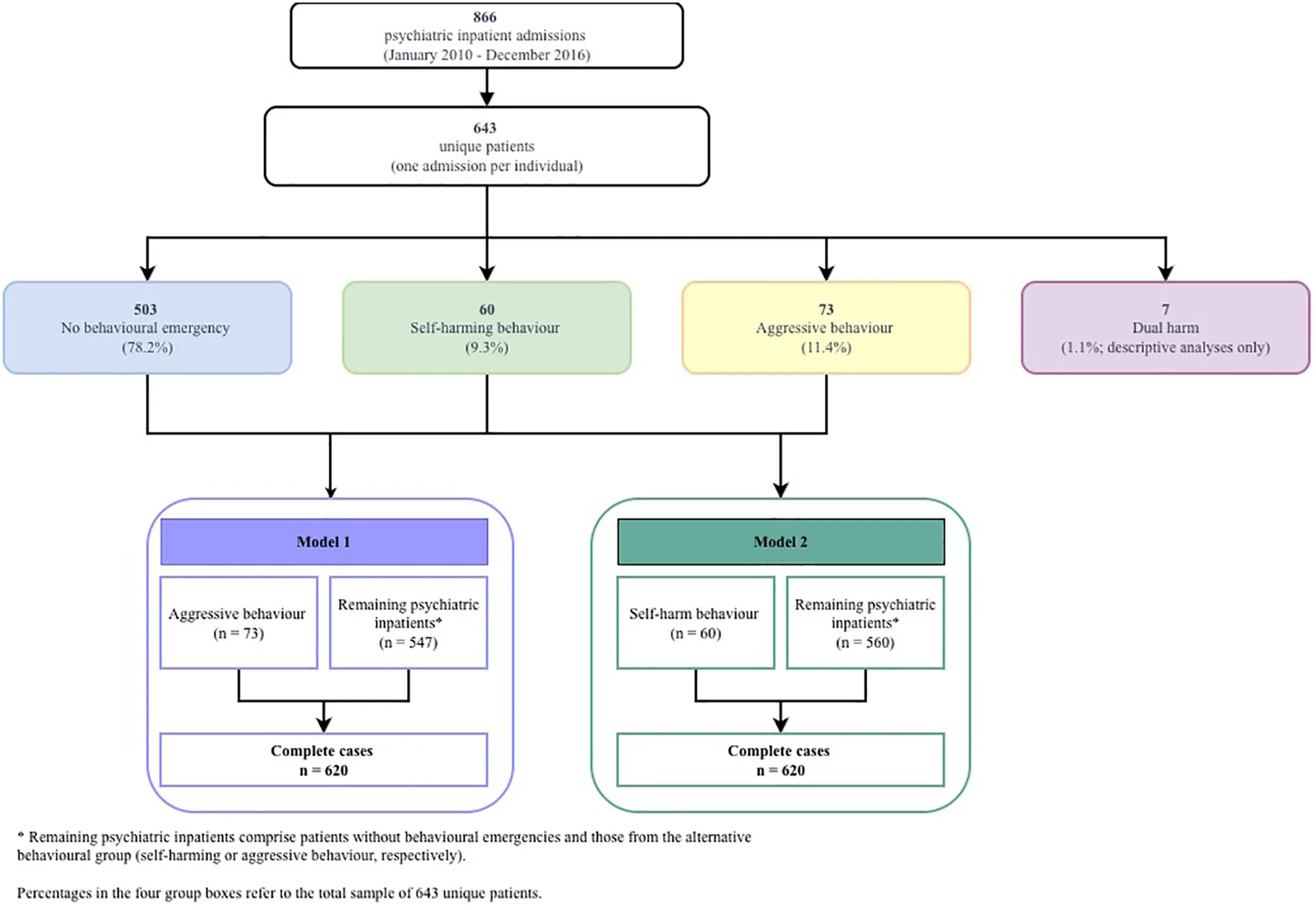
Participant flow and analytic samples.

Patients were classified into four mutually exclusive groups according to their behavioural presentation during the index admission: 503 patients (78.2%) without documented behavioural emergencies, 60 (9.3%) with self-harming behaviour, 73 (11.4%) with aggressive behaviour, and 7 (1.1%) with co-occurring aggressive and self-harming behaviour (dual harm). Because only seven patients presented with dual harm, this group was included in the descriptive analyses but excluded from comparative analyses owing to insufficient statistical power. The participant selection process is illustrated in **Figure 1**.

### Baseline characteristics

Baseline characteristics according to behavioural presentation are summarised in **Table 1**.

**Table 1:** Baseline characteristics according to behavioural presentation.

Characteristic	Other psychiatric inpatients (n = 503)	Self-harming behaviour (n = 60)	Aggressive behaviour (n = 73)	p^a^	p^b^
Sociodemographic variables					
Age, years, mean ± SD	38.4 ± 11.9	36.8 ± 11.4	37.5 ± 11.7	0.082	0.151
German nationality, n (%)	303 (60.7)	27 (45.0)	43 (60.6)	**0.026**	1.000
Interpreter required, n (%)	30 (6.0)	6 (10.0)	5 (6.8)	0.313	0.856
In a relationship, n (%)	43 (8.9)	12 (20.3)	2 (3.0)	**0.011**	0.149
Parenthood, n (%)	149 (30.4)	26 (46.4)	8 (11.6)	**0.009**	**0.020**
Detention-related variables					
Remand detention, n (%)	99 (19.7)	18 (30.5)	19 (26.0)	0.062	0.217
>5 previous imprisonments, n (%)	35 (7.1)	5 (8.6)	11 (15.7)	0.062	**0.032**

SD, standard deviation.

^a^ Other psychiatric inpatients vs. self-harming behaviour.

^b^ Other psychiatric inpatients vs. aggressive behaviour.

Continuous variables were compared using independent-samples t-tests. Categorical variables were compared using two-sided Fisher’s exact tests.

Bold values indicate statistically significant results at p < 0.05.

Compared with other psychiatric inpatients, patients presenting with self-harming behaviour were significantly more likely to be of non-German nationality (*p* = 0.026), to report being in a relationship (*p* = 0.011), and to have children (*p* = 0.009). No significant differences were observed with regard to age, interpreter requirement, detention status, or a history of more than five previous imprisonments.

Compared with other psychiatric inpatients, patients presenting with aggressive behaviour were significantly less likely to have children (*p* = 0.020) and more likely to have a history of more than five previous imprisonments (*p* = 0.032). No significant differences were observed with regard to age, nationality, interpreter requirement, relationship status, or detention status.

### Primary psychiatric diagnoses

Primary psychiatric diagnoses are summarised in **Table 2**.

**Table 2:** Primary psychiatric diagnoses according to behavioural presentation.

Primary psychiatric diagnosis	Other psychiatric inpatients (n = 503)	Self-harming behaviour (n = 60)	Aggressive behaviour (n = 73)	p^a^	p^b^
Organic mental disorders	10 (2.0)	0 (0.0)	0 (0.0)	0.401	0.377
Substance use disorders	33 (6.6)	7 (11.7)	2 (2.7)	0.177	0.294
Schizophrenia spectrum disorders	244 (48.5)	15 (25.0)	53 (72.6)	**<0.001**	**<0.001**
Mood disorders	32 (6.4)	5 (8.3)	7 (9.6)	0.579	0.317
Stress-related disorders	143 (28.4)	25 (41.7)	8 (11.0)	**0.038**	**0.002**
Personality disorders	24 (4.8)	7 (11.7)	3 (4.1)	**0.037**	1.000
Intellectual disability	3 (0.6)	0 (0.0)	0 (0.0)	1.000	1.000
Behavioural and emotional disorders	6 (1.2)	0 (0.0)	0 (0.0)	0.634	1.000

^a^Other psychiatric inpatients vs. self-harming behaviour.

^b^Other psychiatric inpatients vs. aggressive behaviour.

Values are presented as n (%). P-values were calculated using two-sided Fisher’s exact tests.

Bold values indicate statistically significant results at p < 0.05.

Compared with other psychiatric inpatients, patients presenting with self-harming behaviour were significantly less likely to have schizophrenia spectrum disorders (ICD-10 F2; *p* < 0.001) and significantly more likely to have stress-related disorders (ICD-10 F4; *p* = 0.038) or personality disorders (ICD-10 F6; *p* = 0.037). No significant differences were observed with regard to the remaining diagnostic categories.

Compared with other psychiatric inpatients, patients presenting with aggressive behaviour were significantly more likely to have schizophrenia spectrum disorders (ICD-10 F2; *p* < 0.001) and significantly less likely to have stress-related disorders (ICD-10 F4; *p* = 0.002). No significant differences were observed with regard to the remaining diagnostic categories.

### Multivariable logistic regression

Independent factors associated with aggressive behaviour and self-harming behaviour were examined using two multivariable logistic regression models. Results are summarised in **Table 3**.

**Table 3:** Multivariable logistic regression analyses of factors associated with behavioural presentation.

Predictor	Aggressive behaviour vs. remaining psychiatric inpatients OR (95% CI)	Self-harming behaviour vs. remaining psychiatric inpatients OR (95% CI)
Age (per year)	1.00 (0.97–1.02)	0.98 (0.95–1.01)
Sentenced imprisonment†	0.65 (0.36–1.18)	0.76 (0.40–1.46)
Parenthood	**0.38 (0.17–0.82)**	**2.13 (1.17–3.88)**
Schizophrenia spectrum disorders	1.93 (0.89–4.18)	**0.42 (0.18–0.97)**
Stress-related disorders	0.56 (0.21–1.53)	1.13 (0.52–2.47)
Personality disorders	1.09 (0.27–4.37)	2.02 (0.70–5.84)
Model fit		
Complete cases	620	620
Hosmer–Lemeshow test (p)	0.563	0.923
Nagelkerke’s R²	0.086	0.086

CI, confidence interval; OR, odds ratio.

†Reference category: remand detention.

Values are presented as odds ratios (ORs) with 95% confidence intervals (95% CIs). All predictors were entered simultaneously using the enter method. Model fit was assessed using the Hosmer–Lemeshow goodness-of-fit test and Nagelkerke’s R².

Bold values indicate statistically significant results at p < 0.05.

Compared with the remaining psychiatric inpatients, parenthood was independently associated with lower odds of aggressive behaviour (OR 0.38, 95% CI 0.17–0.82, *p* = 0.015). No other variables remained independently associated with aggressive behaviour after adjustment.

Similarly, compared with the remaining psychiatric inpatients, parenthood was independently associated with higher odds of self-harming behaviour (OR 2.13, 95% CI 1.17–3.88, *p* = 0.013), whereas schizophrenia spectrum disorders were independently associated with lower odds of self-harming behaviour (OR 0.42, 95% CI 0.18–0.97, *p* = 0.043). No other variables remained independently associated with self-harming behaviour after adjustment.

## Discussion

The present study compared psychiatric inpatients presenting with aggressive or self-harming behavioural emergencies with other psychiatric inpatients within a specialised prison hospital. Three principal findings emerged. First, schizophrenia spectrum disorders were independently associated with lower odds of self-harming behaviour after multivariable adjustment. Second, although stress-related disorders and personality disorders were more frequent among patients presenting with self-harming behaviour in the descriptive analyses, these associations did not remain significant after multivariable adjustment. Third, parenthood was independently associated with lower odds of aggressive behaviour and higher odds of self-harming behaviour.

### Schizophrenia spectrum disorders and aggression

Schizophrenia spectrum disorders were independently associated with lower odds of self-harming behaviour after multivariable adjustment. Although schizophrenia spectrum disorders were more common among patients presenting with aggressive behaviour, this association did not remain statistically significant after adjustment in the comparison with the remaining psychiatric inpatient population.

The present findings extend previous work from the Berlin Prison Hospital. Using the same underlying clinical cohort, Seidel et al. (20) identified schizophrenia spectrum disorders as an independent correlate of violent behaviour. By analysing patients rather than admissions, the present study enabled a patient-level assessment of behavioural emergencies among psychiatric inpatients treated in a specialised prison hospital.

The observed association between schizophrenia spectrum disorders and aggressive behaviour is consistent with findings from the broader psychiatric literature. Meta-analyses have demonstrated an increased risk of interpersonal violence among individuals with schizophrenia compared with the general population. However, this association is substantially influenced by active psychotic symptoms, comorbid substance use disorders, previous violence, treatment adherence, and adverse social circumstances rather than schizophrenia itself (21–23). Accordingly, schizophrenia should not be interpreted as a direct cause of aggressive behaviour. Rather, the present findings most likely reflect the characteristics of a highly selected population of acutely ill psychiatric inpatients requiring specialised treatment within a correctional setting.

The finding that schizophrenia spectrum disorders were associated with lower odds of self-harming behaviour should also be interpreted cautiously. Given the retrospective observational design of the present study, this association should not be interpreted as indicating a protective effect. Rather, it suggests that, within this population of acutely ill prison psychiatric inpatients, schizophrenia spectrum disorders may differ in their associations with aggressive and self-harming behavioural emergencies in this population.

### Stress-related disorders and personality disorders

Patients presenting with self-harming behaviour more frequently had stress-related disorders and personality disorders in the descriptive analyses. However, neither diagnostic category remained independently associated with behavioural presentation after multivariable adjustment.

The descriptive findings are consistent with previous prison research linking self-harming behaviour to trauma-related psychopathology, emotional dysregulation, and personality pathology (2, 24). Furthermore, systematic reviews have identified previous self-harm, current psychiatric disorders, depression, borderline personality disorder, and adverse prison experiences as major risk factors for self-harm among incarcerated individuals (2, 25).

The loss of these associations after multivariable adjustment suggests that the observed descriptive differences may reflect shared vulnerability factors rather than diagnosis-specific effects. Psychiatric disorders rarely occur in isolation among incarcerated individuals, and substantial psychiatric comorbidity is characteristic of correctional psychiatric populations (26). Consequently, stress-related disorders and personality disorders may represent markers of broader clinical complexity rather than independent determinants of behavioural presentation. This interpretation is consistent with contemporary biopsychosocial models of behavioural emergencies in prison, which emphasise that behavioural crises arise through the interaction of psychiatric vulnerability with psychological, social, and environmental factors rather than from individual psychiatric diagnoses alone (2, 7).

### Parenthood

An unexpected finding of the present study was the independent association between parenthood and behavioural presentation. Patients with children had lower odds of presenting with aggressive behaviour, whereas parenthood was independently associated with higher odds of self-harming behaviour after multivariable adjustment.

To our knowledge, parenthood has received little attention as a potential correlate of behavioural emergencies in correctional psychiatric populations. However, growing evidence indicates that social connectedness and family relationships are important aspects of prisoners’ mental health. Recent systematic reviews have identified reduced social support, loneliness, and limited family contact as important risk factors for self-harming behaviour during imprisonment, whereas supportive interpersonal relationships may mitigate psychological distress (2, 7, 8). Similarly, a recent study conducted in a specialised prison psychiatric unit highlighted the importance of social and environmental factors in understanding suicidal behaviour among incarcerated psychiatric patients (18).

At first glance, the present findings appear counterintuitive, as parenthood might be expected to reflect greater social connectedness. However, parenthood does not necessarily indicate the presence of supportive family relationships. Previous research has shown that separation from children, disrupted family contact, parenting-related stress, and concerns about parental responsibilities are important sources of psychological distress among incarcerated parents and are associated with depression, anxiety, and institutional adjustment difficulties during imprisonment (26). Because information on family functioning, parent–child relationships, custody arrangements, and frequency of family contact was not available, the mechanisms underlying this association remain uncertain. Future research should therefore examine whether the observed association reflects differences in social connectedness, family-related stress, or other unmeasured psychosocial factors rather than parenthood itself.

### Clinical implications

The present findings suggest that aggressive behaviour and self-harming behaviour are associated with different clinical correlates among psychiatric inpatients in specialised prison care. While schizophrenia spectrum disorders were associated with aggressive behaviour in the descriptive analyses, only the inverse association with self-harming behaviour remained statistically significant after multivariable adjustment.

These findings highlight the importance of comprehensive psychiatric assessment that considers not only psychiatric diagnoses but also psychosocial and environmental factors. Routinely available sociodemographic characteristics, such as parenthood, may help identify patients who warrant a more detailed assessment of social connectedness and family-related stress. Prevention and management strategies should therefore integrate evidence-based psychiatric treatment with interventions targeting psychosocial vulnerability, social connectedness, and suicide prevention.

### Future directions

Future studies should investigate behavioural emergencies in correctional psychiatric populations using longitudinal designs and standardised clinical assessments. In particular, further research is needed to clarify the mechanisms underlying the observed association between parenthood and behavioural presentation by incorporating measures of family relationships, social connectedness, and parent–child contact. Such studies may help determine whether the observed associations reflect broader psychosocial vulnerability rather than parenthood itself.

Future research should also examine individuals with dual harm, as recent evidence suggests that the co-occurrence of aggression and self-harming behaviour may represent a clinically important subgroup requiring further investigation (12, 13).

### Strengths and limitations

This study has several limitations. First, its retrospective single-centre design precludes causal inference and may limit the generalisability of the findings beyond comparable correctional psychiatric services. Because all participants were recruited from a specialised prison psychiatric hospital, variability in some sociodemographic and clinical characteristics may have been limited, potentially reducing the ability to detect smaller between-group differences.

Second, behavioural presentations were derived from routine clinical documentation rather than prospectively standardised assessments, and some degree of misclassification cannot therefore be excluded.

Third, although all eligible patients over a seven-year period were included, the relatively small number of patients presenting with aggressive or self-harming behaviour limited statistical power, particularly for subgroup analyses. To minimise the risk of model overfitting, multivariable analyses were restricted to a small number of clinically relevant variables selected *a priori* based on previous literature, clinical relevance, and the observed univariable findings. Consequently, other potentially important factors, including drug misuse, previous violent or self-harming behaviour, nationality, and less frequent diagnostic categories, could not be included in the regression models. Residual confounding by unmeasured variables therefore cannot be excluded. In addition, the relatively small complete-case sample may have limited model stability, and complete-case analysis may have introduced bias if missing data were not completely at random.

Nevertheless, the study also has several important strengths. All consecutive psychiatric admissions to the only specialised prison hospital providing inpatient psychiatric care in the federal state of Berlin over a seven-year period were considered for inclusion. To our knowledge, this is the first patient-level study to investigate aggressive and self-harming behavioural emergencies among psychiatric inpatients treated in a specialised prison hospital. By extending the previous work of Seidel et al. (20) through patient-level analyses, the present study contributes to a more differentiated understanding of behavioural emergencies in correctional psychiatry and may help inform future risk assessment and prevention strategies.

## Data Availability

The raw data supporting the conclusions of this article will be made available by the authors, without undue reservation.
